# A review of the ketogenic diet for endurance athletes: performance enhancer or placebo effect?

**DOI:** 10.1186/s12970-020-00362-9

**Published:** 2020-06-22

**Authors:** Caitlin P. Bailey, Erin Hennessy

**Affiliations:** The Gerald J. and Dorothy R. Friedman School of Nutrition Science and Policy at Tufts University, 150 Harrison Avenue, Boston, MA 02111 USA

**Keywords:** Ketogenic diet, High fat diet, Ketosis, Endurance athlete, VO_2_ max

## Abstract

**Background:**

The ketogenic diet has become popular among endurance athletes as a performance enhancer. This paper systematically reviews the evidence regarding the effect of the endurance athlete’s ketogenic diet (EAKD) on maximal oxygen consumption (VO_2_ max) and secondary performance outcomes.

**Methods:**

PubMed and Web of Science searches were conducted through November 2019. Inclusion criteria were documentation of EAKD (< 50 g daily carbohydrate consumed by endurance athletes), ketosis achieved (measured via serum biomarker), VO_2_ max and/or secondary outcomes, English language, and peer reviewed-publication status. Articles were excluded if they were not a primary source or hypotheses were not tested with endurance athletes (i.e., individuals that compete at submaximal intensity for extended time periods). Study design, diet composition, adherence assessment, serum biomarkers, training protocols, and VO_2_ max/secondary outcomes were extracted and summarized.

**Results:**

Searches identified seven articles reporting on VO_2_ max and/or secondary outcomes; these comprised six intervention trials and one case study. VO_2_ max outcomes (*n* = 5 trials, *n* = 1 case study) were mixed. Two of five trials reported significant increases in VO_2_ max across all diets; while three trials and one case study reported no significant VO_2_ max findings. Secondary outcomes (*n* = 5 trials, *n* = 1 case study) were Time to Exhaustion (TTE; *n* = 3 articles), Race Time (*n* = 3 articles), Rating of Perceived Exertion (RPE; *n* = 3 articles), and Peak Power (*n* = 2 articles). Of these, significant findings for EAKD athletes included decreased TTE (*n* = 1 article), higher RPE (*n* = 1 article), and increased Peak Power (*n* = 1 article).

**Conclusion:**

Limited and heterogeneous findings prohibit definitive conclusions regarding efficacy of the EAKD for performance benefit. When compared to a high carbohydrate diet, there are mixed findings for the effect of EAKD consumption on VO_2_ max and other performance outcomes. More randomized trials are needed to better understand the potentially nuanced effects of EAKD consumption on endurance performance. Researchers may also consider exploring the impact of genetics, recovery, sport type, and sex in moderating the influence of EAKD consumption on performance outcomes.

## Background

The ketogenic diet prescribes a significant reduction in carbohydrate intake, which facilitates physiological changes that promote the utilization of ketones [1]. Recently this diet has received attention from the endurance community as a potential ergogenic aid because it minimizes the body’s reliance on carbohydrates. Despite evidence-based guidance for athletes to consume adequate carbohydrates [2], it has been proposed that the biological constraints of carbohydrate storage may limit athletes who compete over extended time periods [3, 4]. Carbohydrates are stored in the body predominately as glycogen in muscle tissue (300 g) and liver tissue (90 g), in addition to glucose in the blood stream (30 g) [5]. This amounts to roughly 1680 kcal of available energy from carbohydrate at any one time. As a result, endurance athletes must replenish their glycogen stores every one to three hours during activity [5]. This continual consumption redirects nutrients from exercising muscles to the gut to aide digestion, potentially leading to reduced exercise economy and digestive disturbances, which compromise the athlete’s ability to maximize training and competition outcomes [3]. Additionally, research indicates that training with low muscle glycogen availability promotes molecular changes that enhance training-derived endurance adaptations [6]. Furthermore, ketogenic diets have been shown to reduce lactate accumulation after exercise, contributing to enhanced recovery [7, 8]. Taken together, this evidence suggests that reduced reliance on carbohydrates via ketosis can produce beneficial results for endurance athletes.

In contrast to the limitations of carbohydrate storage, the body can reserve large amounts of energy in the form of fat. One pound of fat yields approximately 3500 kcal, making fat a vast source of energy, even among relatively lean endurance athletes. In theory, if endurance athletes tolerate the ketogenic diet, they could achieve longer training periods with sustained energy levels and reduced need for refueling, allowing them to maximize the aerobic benefits from training and competing. In fact, there is some evidence that, among highly trained individuals, benefits of the diet include a steady supply of energy for the body and brain during prolonged exercise and accelerated recovery time post-exercise [4]. While scientists continue to explore potential benefits and drawbacks of the endurance athlete’s ketogenic diet (EAKD), several public figures in the athletic community have already embraced the diet as ergogenic [9, 10]. However, to the authors’ knowledge, there have been no systematic reviews of EAKD consumption and endurance outcomes (e.g., VO_2_ max, TTE, Race Time, RPE, Peak Power) from which such conclusions may be drawn.

To fill this gap, the present review characterizes the nature and extent of available scientific evidence regarding the claim that EAKD consumption results in improved endurance performance, as measured by maximal oxygen uptake (VO_2_ max). VO_2_ max is considered the gold standard for measuring aerobic fitness. It is measured via a graded exercise test on a treadmill or a cycle ergometer, and quantified as the body’s maximum oxygen use in milliliters per kilogram of body weight per minute [11]. Higher levels of VO_2_ max indicate greater endurance capacity. It is important to note that while VO_2_ max is an established measure of endurance capacity, relative VO_2_ max is confounded by changes in body weight and thus not without limitations. For this reason, secondary performance outcomes (i.e., time to exhaustion [TTE], race time, rating of perceived exertion [RPE], peak power) were also collected for analysis.

This manuscript is intended to enhance the athletic and scientific communities’ knowledge of the potential benefits and consequences of adopting the EAKD, and to identify gaps in the current literature that may create opportunities for future study. Specifically, this review focuses on peer-reviewed articles examining endurance athletes (e.g., cyclists, runners, race walkers, triathletes) participating in three or more weeks of EAKD consumption. The included studies looked at a variety of outcomes; however, the primary outcome of interest to this review is VO_2_ max.

## Main text

### Methods

Articles were identified for inclusion via electronic database literature searches. An initial search was conducted using Web of Science and PubMed, on February 1, 2018. Subsequent searches of Web of Science and PubMed were conducted, using identical search criteria, in order to capture the most recent publications available. The final search was conducted on November 17, 2019. The following key terms were used to search the databases for articles by topic: *ketogenic, race, walker, cyclist, runner, marathon, endurance,* and *athlete*. The full search strategy used for both databases is as follows: ((ketogenic) AND (race[Title] OR walker*[Title] OR cyclist*[Title] OR runner*[Title] OR marathon*[Title] OR endurance[Title] OR athlet*[Title])). Asterisks denote truncation. Additional inclusion criteria were English language, peer reviewed-publication status, ketosis achieved (as measured via serum biomarkers), and documentation of VO_2_ max and/or secondary outcomes. The following exclusions were applied to the searches in order to narrow the scope of the article lists generated: NOT (epilepsy or child or mice or mouse or diabet* or rat* or seizure).

Articles were included for review if the title, abstract, or key words indicated that the study focused on the ketogenic diet in the context of endurance sport training and/or racing (i.e., the EAKD). Articles that met inclusion criteria from each database were compiled using Endnote software. Duplicates were removed, and abstracts were pre-screened for source type. Articles were excluded if they were not a primary source.

After identifying all eligible records, a data matrix was developed and data were extracted on the following variables: study design, athlete type (i.e., sport, training level, age range), diet type (i.e., EAKD, high carbohydrate, periodised carbohydrate) and composition, recruitment numbers, study length, dietary adherence assessment method, serum biomarkers for ketosis, training protocols, and VO_2_ max/secondary outcomes. Data from the matrix are presented in Tables 1 and 2. Results were synthesized qualitatively.

**Table 1:** Descriptive results

Reference	Sample size	Population, age range	Study design	Study length	Methods				
					Diet composition	Diet provision & assessment	Ketosis biomarker	Training protocol	VO_**2**_ Max protocol
**Prospective trials**									
Burke et al. 2017 [12]	*N* = 29	Professional male race walkers with international race experience, 21–32 years	Self-selected diet (non-random assignment)	3 weeks	Diets:**EAKD** (< 50 g CHO, 75–80% FAT, 15–20% PRO [*n* = 10])^a^; **HCD** (60–65% CHO, 20% FAT, 15–20% PRO [*n* = 9])^a^; **PCHO** (60–65% CHO, 20% FAT, 15–20% PRO [*n* = 10])^a^	Personalized menus developed by professional chef and RDs. All foods provided/recorded by research team.	Beta-hydroxybutyrate levels post-EAKD: 0.8–2.0 mmol/liter	Olympic-level training camp. Included daily race walking, resistance training, and/or cross training.	Treadmill test
Carr et al. 2018 [7]	*N* = 24	Male (*n* = 17) and female (*n* = 7) elite race walkers	Self-selected diet (non-random assignment)	3 weeks	Diets:**EAKD** (< 50 g CHO, 75–80% FAT, 15–20% PRO [*n* = 9]); **HCD** (60–65% CHO, 20% FAT, 15–20% PRO [*n* = 8]); **PCHO** (60–65% CHO, 20% FAT, 15–20% PRO [*n* = 7])	Menus developed by professional chef and RDs. All foods provided/recorded by research team.	Elevated serum ketone bodies post-EAKD: 1 mmol/liter	Supervised, sport-specific, 3-week training protocol.	Treadmill test
Heatherly et al. 2018 [13]	*N* = 8	Middle-age, recreationally competitive male runners, 39.5 ± 9.9 years	Pre-posttest	3 weeks	Diets:**EAKD** (< 50 g CHO, target 70% FAT [ad libitum]); **HCD** (habitual pre-study diet, reported as “moderate to high CHO”)	Participants provided with daily macronutrient targets and instructed to self-track diet using diet software.	Elevated serum ketone bodies post-dietary intervention compared to pre-EAKD levels: 0.7 ± 0.52 mmol/liter (EAKD) vs. 0.25 ± 0.09 mmol/liter (CHO)	Participants continued normal recreational athletic activity for study duration.	Treadmill test (pre-EAKD only). % baseline VO_2_ max at various race paces post-EAKD reported.
McSwiney et al. 2018 [14]	*N* = 20	Male endurance trained athletes (e.g., triathlon, cycling, marathon, ultra-marathon), 18–40 years	Self-selected diet (non-random assignment)	12 weeks	Diets:**EAKD** (< 50 g CHO, > 75% FAT, 10–15% PRO [*n* = 9]); **HCD** (65% CHO, 20% FAT, 14% PRO [*n* = 11])	Participants received detailed handouts (e.g., example meal plans, shopping lists), nutrition counseling, and weekly check-ins. Weekly weighed food diary submitted.	Beta-hydroxybutyrate levels post-EAKD: 0.5 mmol/liter	≥ 7h hours of endurance exercise and 2 strength training sessions per week.	Cycle ergometer test
Phinney et al. 1983 [15]	*N* = 5	Elite male cyclists, 20–30 years	Pre-posttest	4 weeks	Diets:**EAKD** (< 20 g CHO, 85% FAT, 15% PRO); **HCD** (1.75 g PRO/kg/day, remainder as 66% CHO and 33% FAT)^a^	Participants received three meals per day. Portions were weighed and intake monitored.	Beta-hydroxybutyrate levels post-EAKD: 1.28 ± 0.35 mmol/liter	Participants were asked to continue normal training, monitored via daily diary.	Cycle ergometer test
Shaw et al. 2019 [16]	*N* = 8	Male endurance trained athletes (*n* = 2 marathoners, *n* = 4 ultra-marathoners, *n* = 2 triathletes), 29.6 ± 5.1 years	Randomized repeated measures crossover study	31-days (4.5 weeks) per condition with a 14- to 21-day washout period	Diets:**EAKD** (< 50 g CHO, 75–80% FAT, 15–20% PRO); **HCD** (43% CHO, 38% FAT, 19% PRO)^a^	Participants received education session with RD, info booklet, personalized menu plan, meal/snack examples, and lifestyle advice. All had daily contact with a registered dietitian for monitoring.	Beta-hydroxybutyrate levels post-EAKD: ≥0.3 mmol/liter by day 3 and ≥ 0.5 mmol/liter by day 7	Participants designed their own 28-day training plan (running and cycling) and were asked to replicate this during each dietary period.	Treadmill test
**Case studies**									
Zinn et al. 2017 [17]	*N* = 5	Recreational athletes involved in competitive endurance sport for 5+ years, 49–55 years	Pilot case study, mixed methods research	10 weeks	Diet:**EAKD** (< 50 g CHO, ad libitum FAT, 1.5 g/kg PRO [*n* = 5])	Participants provided with daily macronutrient prescription and instructed to self-track diet using diet software.	Beta-hydroxybutyrate levels: 0.5–4.2 mmol/liter	Participants continued normal recreational athletic activity for study duration.	Cycle ergometer test

*EAKD* Endurance Athlete Ketogenic Diet, *HCD* High Carbohydrate Diet, *PCHO* Periodised carbohydrate diet: percentages based on weekly rather than daily diet, *CHO* Carbohydrate, *PRO* Protein, *RD* Registered dietitian

^a^Isocaloric diets

**Table 2:** Study outcomes: VO_2_ max and secondary outcomes. Dashes indicate that studies did not assess the specified variable(s)

Reference	VO_**2**_ max outcomes (mL/kg/min)	Time to exhaustion (TTE)	Race time/Time trial	Rating of perceived exertion (RPE)	Peak power
**Prospective Trials**					
Burke et al. 2017 [12]	**Significant increase in VO**_**2**_**max from baseline (p < 0.001) in all three groups.** VO_2_ Max of the HCD group was significantly lower than for the other groups both pre- and post-diet (p ≤ 0.02). Pre- vs. post-intervention EAKD: 66.3 vs. 71.1 HCD: 61.6 vs. 66.2 PCHO: 64.9 vs. 67.0	**_ _**	EAKD group: Non-significant increase in 10 km race time from baseline. **HCD and PCHO groups: Significant decrease in race time (p < 0.01).** Pre- vs. post-intervention EAKD: 23 s slower HCD: 190 s faster PCHO: 124 s faster	**EAKD group: Significantly higher RPE values for post-intervention graded economy test compared with pre-intervention RPE values (*p*** **≤ 0.01).** Non-significant trend for higher RPE values during 25 km long walk for both pre- and post-testing.	**_ _**
Carr et al. 2018 [7]	**Significant increase in VO**_**2**_**max from baseline (*p*** **< 0.05) in all three groups.** Between groups analysis not reported. Pre- v. post-intervention (M ± SD) EAKD: 61.1 ± 5.3 vs. 63.4 ± 4.1 HCD: 57.6 ± 4.6 vs. 58.3 ± 4.1 PCHO: 58.1 ± 3.3 vs. 60.2 ± 3.8	**_ _**	**_ _**	**_ _**	**_ _**
Heatherly et al. 2018 [13]	Post-EAKD VO_2_ max not measured. Study reported % baseline VO_2_ max at various race paces. **At 10 km, 21 km, 42 km and sub-42 km (but not 5 km) race paces, % relative VO**_**2**_**max was significantly greater post-EAKD.** Example (10 km pace; p < 0.05): EAKD: 98.7 ± 11.3 HCD: 92.8 ± 5.3	**_ _**	5 km time trial time was not significantly different pre- vs. post-EAKD (*p* > 0.10). Pre- vs. post-intervention EAKD: 23.45 ± 2.25 min. HCD: 23.92 ± 2.57 min.	Overall RPE did not differ significantly pre- vs. post-EAKD during 5 km time trial (*P* > 0.10). Pre- vs. post-intervention EAKD: 8.4 ± 1.2 HCD: 8.0 ± 1.0	**_ _**
McSwiney et al. 2018 [14]	Increase in both groups post-diet. Non-significant difference between groups (*p* = 0.968). Pre- vs. post-intervention EAKD: 53.6 ± 6.8 vs. 57.3 ± 6.7 HCD: 52.6 ± 6.4 vs. 57.2 ± 6.1	**_ _**	100 km time trial time was not significantly different between groups (*p* = 0.057). Pre- vs. post-intervention EAKD: 4.07 min.sec faster HCD: 1.13 min.sec faster	**_ _**	**Post-intervention peak power was significantly different between groups (*p*** **= 0.047).** Pre- vs. post-intervention EAKD: 8.3 ± 2.2 vs. 9.7 ± 2.3; 1.4 watts/kg increase HCD: 9.1 ± 2.6 vs. 8.4 ± 2.2; 0.7 watts/kg decrease
Phinney et al. 1983 [15]	Non-significant decrease from baseline (HCD; *p* > 0.01). Pre- vs. post-intervention EAKD: 5.00 ± 0.20 HCD: 5.10 ± 0.18	Non-significant increase in mean exercise times from baseline (HCD). Pre- vs. post-intervention EAKD: 151 ± 25 min. HCD: 147 ± 13 min.	**_ _**	**_ _**	**_ _**
Shaw et al. 2019 [16]	No significant change from pre-intervention levels for either dietary exposure (*p* > 0.05). Pre-intervention (all athletes) 59.4 ± 5.2	No significant difference between dietary interventions (*p* = 0.56). Pre- vs. post-intervention EAKD: 239 ± 27 vs. 219 ± 53 min. (*p* = 0.36) HCD: 237 ± 44 vs. 231 ± 35 min. (*p* = 0.44)	**_ _**	RPE values were similar for each dietary intervention during run-to-exhaustion trials. 1-h, 2-h, at exhaustion EAKD: 11.4 ± 0.9, 12.1 ± 1.4, 19.38 ± 0.52 HCD: 11.7 ± 0.8, 12.8 ± 0.9, 19.38 ± 0.52	**_ _**
**Case studies**					
Zinn et al. 2017 [17]	Non-significant change from baseline (M ± SD): − 1.69 ± 3.4 (*p* = 0.63). (with a decrease in four of the five athletes)	**Significant decrease in TTE for all participants (*p*** **= 0.004).** Mean change from baseline EAKD: − 2 ± 0.7 min.	**_ _**	**_ _**	Four out of five athletes experienced a decrease in peak power from baseline (*p* = 0.07). Mean change from baseline EAKD: − 18 ± 16.4 watts

*EAKD* Endurance Athlete Ketogenic Diet, *HCD* High Carbohydrate Diet, *PCHO* Periodised carbohydrate diet

## Results

### Search results

**Fig. 1:**
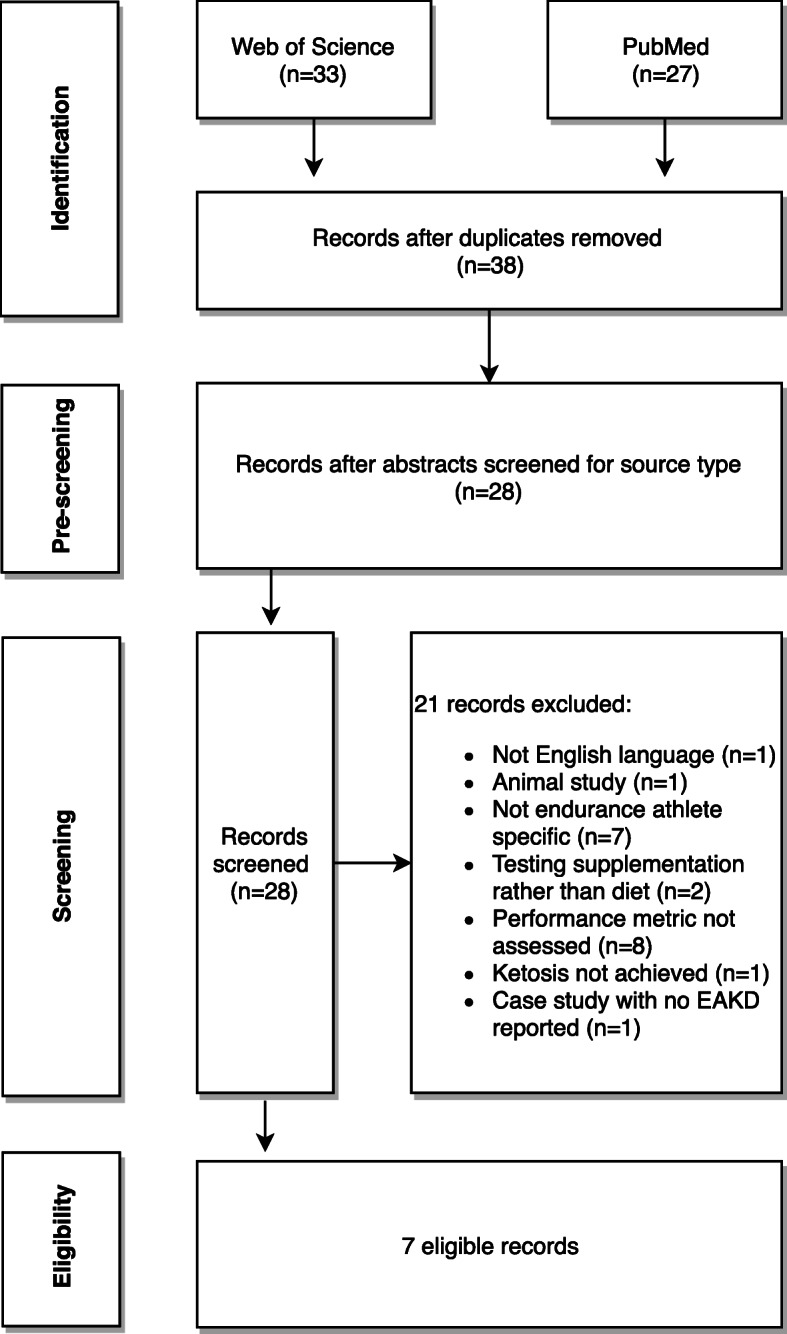
Flow chart depicting the literature search and review process to arrive at the final analytic sample (*n* = 7). Arrows pointing right indicate the number of articles excluded and for what reason

Figure 1 illustrates the screening process and articles included in this review. In brief, searches from Web of Science and PubMed generated *n* = 60 articles (*n* = 33 and 27, respectively). After removing duplicates and pre-screening, 28 articles remained. After further review, 21 additional records were excluded (see Fig. 1 for reasons for exclusion). All exclusions were conducted to emphasize the effect of ketogenic diet consumption on sport-specific performance in endurance athletes. The screening process produced seven eligible articles: six prospective trials (*n* = 1 randomized crossover study, *n* = 3 non-randomized trials, *n* = 2 pre-posttest), and one case study. See Fig. 1 for a flow chart of the screening process.

### Descriptive results

Among the seven studies included in this review, sex and athlete type were inextricable variables. Five of seven studies examined VO_2_ max outcomes in only male athletes [12–16]. However, among those studies, athlete type varied: one study recruited male runners [13], one recruited male race walkers [12], one recruited male cyclists [15], and two recruited a mixed sample of male endurance athletes [14, 16]. Two of the seven studies recruited both male and female athletes; one recruited a sample of race walkers [7] and the other recruited a sample of mixed endurance athletes [17]. Ages for study participants ranged from 18 to 55 years. All seven studies included an EAKD (< 50 g daily carbohydrate). Of the six trial studies, all included a standard, high carbohydrate comparison diet [7, 12–16], while the case study provided no comparison diet [17]. Studies either provided participants with meals [7, 12, 15] or with dietary guidance, including sample meal plans [13, 14, 16, 17]. Adherence to diet was tracked via objective researcher observation and measurement [7, 12, 15] or participant self-report (e.g., weighed food diaries, dietary analysis software) [13, 14, 16, 17]. All studies explicitly reported tracking serum ketone levels as a biomarker for ketosis. All studies lasted between three and 12 weeks.

### Performance outcome results

VO_2_ max outcomes (mL/kg/min; *n* = 6 studies) were mixed: two studies reported significant increases in VO_2_ max across all diets [7, 12], and four reported no significant VO_2_ max outcomes [14–17]. In a three-week nonrandomized trial, Carr et al. reported significant increases in VO_2_ max from baseline for all diet types (EAKD: 61.1 ± 5.3 vs. 63.4 ± 4.1; HCD: 57.6 ± 4.6 vs. 58.3 ± 4.1; PCHO: 58.1 ± 3.3 vs. 60.2 ± 3.8; *p* < 0.05) [7]. Using a similar design, Burke et al. found a significant increase in VO_2_ max for all athletes (EAKD: 66.3 vs. 71.1; HCD: 61.6 vs. 66.2; PCHO: 64.9 vs. 67.0; *p* < 0.001) [12]. McSwiney et al. showed a 3.7-unit increase in relative VO_2_ max among the EAKD group after 12 weeks (53.6 ± 6.8 vs. 57.3 ± 6.7) [14]. This was a smaller increase than the 4.6-unit increase observed in the comparison diet group (52.6 ± 6.4 vs. 57.2 ± 6.1); furthermore, the increase in relative VO_2_ max during EAKD consumption was inflated by a 6-kg mean reduction in body mass among the participants. The difference in increase between the two groups was not significant (*p* = 0.968) [14]. Shaw et al., a randomized crossover study, found no significant changes in VO_2_ max from baseline (59.4 ± 5.2) after either 31 days of EAKD or high carbohydrate comparison diet (*p* > 0.05) [16]. Using a pre-posttest design, Phinney et al. found no difference in VO_2_ max between a high carbohydrate comparison diet and EAKD (pre-intervention HCD: 5.10 ± 0.18; EAKD: 5.00 ± 0.20; *p* > 0.01) [15]. Heatherly et al., also a pre-posttest design, measured VO_2_ max pre- but not post-EAKD consumption [13]. Instead, this study reported on the percent of baseline (pre-dietary intervention) VO_2_ max achieved at various race paces tested post-EAKD consumption. Researchers found that the percent of baseline relative VO_2_ max achieved was significantly greater post-EAKD at 10 km, 21 km, 42 km, and sub-42 km (but not 5 km) race paces (see Table 2; *p* < 0.05) [13]. Finally, Zinn et al. showed a non-significant decrease from baseline VO_2_ max in athletes consuming the EAKD after 10 weeks (− 1.69 ± 3.4; *p* = 0.63) [17]. Zinn et al. was a case study with no reference comparison diet.

Secondary outcomes (*n* = 6 studies) were also mixed. Of three studies that reported TTE, Shaw et al. and Phinney et al. each found no significant difference in TTE by diet type [15, 16], while Zinn et al. reported a significant decrease from baseline (pre-dietary intervention) for all five case study participants consuming the EAKD (− 2 ± 0.7 min.; *p* = 0.004) [17]. Differences in race times by dietary intervention were reported by three studies [12–14] and found to be significant in one [12]. Specifically, Burke et al. reported a significant decrease in race time among high carbohydrate and periodized carbohydrate groups (HCD: − 190 s; PCHO: − 124 s; *p* < 0.01), while the EAKD group had a non-significant increase in race time (EAKD: + 23 s; *p* > 0.01) [12]. RPE was measured in three studies [12, 13, 16] and found to be significantly different from baseline in one [12]. Burke et al. reported higher RPE values among the EAKD group post-intervention compared with pre-intervention (*p* ≤ 0.01) [12]. Finally, peak power was measured in two studies [14, 17]. McSwinney et al. reported that post-intervention peak power was significantly different between diets, with EAKD athletes improving their peak power and comparison diet athletes decreasing their peak power (EAKD: 8.3 ± 2.2 vs. 9.7 ± 2.3 watts/kilogram; HCD: 9.1 ± 2.6 vs. 8.4 ± 2.2 watts/kilogram; *p* = 0.047). Zinn et al. found a mean decrease in peak power from baseline (− 18 ± 16.4 watts; *p* = 0.07) with a decrease in four out of five athletes [17]. See Table 2 for a full list of results.

## Discussion

It has been hypothesized that consuming a ketogenic diet may enhance performance among endurance athletes by promoting a shift in substrate utilization that enhances physiological training benefits [3, 18]. The present review explores this hypothesis by examining associations between EAKD consumption and VO_2_ max, a biomarker for endurance capacity [11]. Two of the seven studies included in this review found a significant increase in VO_2_ max post-EAKD consumption [7, 12]. However, both articles reported significant VO_2_ max increases across all diets, and that outcomes were independent of dietary intervention. Interestingly, both studies were conducted among elite race walkers that self-selected their dietary intervention, and the athletes that self-selected into the EAKD had slightly higher average baseline and post-treatment VO_2_ max values [7, 12]. Furthermore, Burke et al., reported that VO_2_ max values for the high carbohydrate comparison group were significantly lower than EAKD or periodised carbohydrate groups at baseline and follow-up (*p* ≤ 0.02) [12]. This suggests that other factors may influence athletes’ choice of diet and aerobic capacity concomitantly, such as genetic variation in trainability and/or chronic substrate utilization [19, 20]. A review conducted by Williams et al. revealed the potential for 97 genes to predict VO_2_ max trainability, suggesting that genetics may account for differing training outcomes among athletes [20]. Certain dietary preferences, which both acutely and chronically influence substrate utilization, have also been linked to gene variations, highlighting the possibility for both dietary choices and training outcomes to be mediated by genetics [19, 21]. Randomized controlled trials and genome-wide association studies can be leveraged to control for, and explore the impact of, such factors in future studies of the EAKD.

Four of the seven studies reviewed reported non-significant VO_2_ max outcomes [14–17]. In a non-randomized trial, McSwiney et al. reported a VO_2_ max increase in both groups of male endurance athletes post-EAKD (EAKD: 53.6 ± 6.8 vs. 57.3 ± 6.7; HCD: 52.6 ± 6.4 vs. 57.2 ± 6.1) with a non-significant difference between groups (*p* = 0.968) [14]. In a pre-posttest design, Phinney et al. reported a non-significant decrease in VO_2_ max from baseline among five elite male cyclists (pre- vs. post-EAKD: 5.10 ± 0.18 vs. 5.00 ± 0.20; *p* > 0.01) [15]. In a case study, Zinn et al. reported a non-significant decrease among five recreational endurance athletes consuming the EAKD (− 1.69 ± 3.4; *p* = 0.63) [17]. Finally, in a randomized crossover study, Shaw et al. reported no significant changes from baseline (59.4 ± 5.2) among male endurance athletes during either dietary intervention (*p* > 0.05) [16].

Heatherly et al. did not report VO_2_ max outcomes, instead providing the percentage of baseline VO_2_ max achieved at various race paces (i.e., 5 km, 10 km, 21 km, 42 km, sub-42 km) [13]. The significantly greater percentages of baseline VO_2_ max achieved post-EAKD consumption at 10 km, 21 km, 42 km, and sub-42 km race paces demonstrate that the EAKD was negatively correlated with the athletes’ aerobic efficiency at these paces. This is corroborated by some of the secondary outcomes reported in Table 2, including reports of EAKD being associated with significantly higher RPE [12], and decreased TTE [17]. Only one study reported significant positive secondary findings: a higher peak power in athletes post-EAKD compared to the standard, high carbohydrate diet [14]. The authors of the study hypothesized that this outcome was likely due to an improved power to weight ratio among the EAKD athletes, who lost an average of 6 kg of body mass.

Despite the popularity of the diet as an ergogenic aid, this review provides evidence that EAKD consumption produces mixed results, in terms of endurance performance, when compared to a high carbohydrate diet. Several biological mechanisms may help to explain the potential for mixed and/or detrimental effects, including changes in fuel economy, production of certain metabolic byproducts, and reduced energy intake. For example, the EAKD significantly increases fat oxidation, requiring greater oxygen consumption due to the increased oxygen demands during fatty acid metabolism versus carbohydrate metabolism [12, 22]. This increased demand for oxygen reduces the beneficial impact of an increased VO_2_ max because a greater percentage of maximal oxygen uptake is now required to maintain any given race pace [13]. Second, EAKD metabolites such as tryptophan and ammonia may promote fatigue by influencing the central nervous system [23, 24]. Finally, it has been shown that the EAKD leads to increased satiety and reduced energy intake [25]. Reduced energy intake, and the accompanying weight loss, may be beneficial for some individuals but could also present a sustainability issue for highly active athletes. Substantial reductions in body weight may negatively impact mental, hormonal, and bone health, as well as recovery time and general exercise performance [26, 27]. Illustrating these mechanisms, Heatherly et al. reported that athletes exhibited greater oxygen consumption at race pace on the EAKD versus a high carbohydrate diet and that ad libitum EAKD consumption resulted in decreased intake of roughly 1000 kcal per day, leading to a 3 % loss of body mass over the study period [13].

In multiple studies, participant self-reports (e.g., interview data, training logs) suggested that the EAKD may have promoted perceived fatigue and decreased ability to train for certain athletes [17], particularly those training in summer months [13]. This could be a combined result of the alterations in fuel economy, metabolism, and energy intake described above, though not all athletes reported experiencing negative side effects. Based on focus group results, one study reported that athletes had more positive than negative perceptions of the diet [17], suggesting that there may be additional unknown variables influencing EAKD outcomes across individuals and/or settings (e.g., temperature, humidity [13]).

One hypothesis for the variation in performance outcomes among studies might stem from the heterogeneity across the training/recovery protocols and fitness levels of the athletes [28]. Both studies exhibiting a statistically significant increase in VO_2_ max examined the effects of EAKD consumption in professional race walkers with high base levels of aerobic capacity, a factor that has been associated with faster recovery times and greater positive adaptations to training [29–31]. Both studies also explicitly included a recovery protocol in their training prescription, which could impact the athletes’ training outcomes [28]. Due to limited information on training/recovery protocols in many of these studies, strong conclusions cannot be generated regarding the impact of training versus diet on performance outcomes. However, based on previous evidence, it is reasonable to hypothesize that these protocol differences may have contributed to the diverse outcomes reported [6, 28, 32].

In examining the results, it is important to bear in mind that this review consists of just seven studies, only one of which was randomized [16]. Carr et al., Burke et al., and McSwiney et al. were all prospective trials, however they allowed participants to choose their dietary intervention [7, 12, 14]. Although this self-selection method generally improves rates of adherence to the diets, it also introduces risk of bias in that those athletes who chose the EAKD may have other lifestyle or dietary tendencies that could affect their biological response to the diet. Heatherly et al. and Phinney et al. were pre-posttest studies, which are subject to threats to internal validity, such as the fact that passage of time results in natural decreases in VO_2_ max [13, 15]. Finally, Zinn et al. was a case study [17]. Although the article provides a wealth of hypothesis generating observations, without a comparison group we cannot conclude whether the EAKD was more or less effective than the standard, high carbohydrate diet for athletes.

All studies had relatively small sample sizes, which reduced the statistical power of the analyses. It is possible that, with a larger sample size, the seven studies might have exhibited corroboratory results. The small sample sizes also exacerbated the problem of drop-out rates, which were considerable in one of the five studies. McSwiney et al. lost 18 participants in the EAKD group and nine in the comparison group, resulting in a participation rate of 33 and 55%, respectively [14].

At the review level, heterogeneity in dietary interventions, adherence measurements, VO_2_ max testing procedures, training protocols, and athlete types all introduced variation that made comparisons across studies difficult. For example, four studies measured VO_2_ max using a treadmill test [7, 12, 13, 16], while the other three studies used a cycle ergometer [14, 15, 17]. Previous reviews suggest that these two testing procedures produce inconsistent results, with higher VO_2_ max outcomes reported for treadmill as compared to cycle ergometer tests [33]. Therefore, inter-article comparisons of the *change* in VO_2_ max by diet from baseline may be more reliable than inter-article comparisons of the absolute outcome values reported. Furthermore, research suggests that VO_2_ max may be an inaccurate predictor of endurance performance in runners, specifically due to variations in running economy and fatigue [34, 35]. Therefore, VO_2_ max may not be a strong indicator of endurance capacity in some sports, further complicating this measure as a comparison across heterogeneous groups of athletes.

In addition to VO_2_ max outcomes, Table 2 provides a matrix of secondary outcomes (i.e., TTE, race time, RPE, peak power), which can be used to complement the VO_2_ max findings from this review. For example, although all three diet groups in the study by Burke et al. experienced a significant increase in VO_2_ max from baseline, only the comparison groups (i.e., high carbohydrate, periodised carbohydrate) experienced faster 10 km race walk times. Furthermore, the EAKD group reported significantly higher RPE values compared to baseline during a graded economy test. Future research in this field can benefit from utilizing a variety of performance metrics, such as the ones discussed in this review, to triangulate overall effects of diet on athletic performance, limiting biases introduced from relying on one marker alone. Additionally, as this research area develops, it may be prudent to conduct reviews among athletes of a single type (e.g., runners only, cyclists only) to limit the heterogeneity among studies.

Because only two databases were used to identify articles for review, it is possible that other studies of EAKD and endurance performance do exist in the literature. However, exploratory investigations of other databases retrieved no additional articles that met inclusion criteria. It is noteworthy that six of seven studies included in this review were published within the last 5 years, suggesting that scientific attention to this topic is fairly recent. Due to the contemporary nature of the research question, it is also possible that yet-to-be-published research exists on this topic. Therefore, future reviews may eventually produce more conclusive evidence. Finally, the potential risk of reporting bias is always present. Unfortunately, it is difficult to assess publication bias because we cannot know the extent of the evidence that has gone unpublished. However, due to the controversial nature of this topic among scientists and lay people alike, it seems likely that both significant and null findings would be publishable.

## Conclusions

Despite popular interest in the ketogenic diet as an ergogenic aid in endurance sport, there are few published studies examining the effect of EAKD consumption on VO_2_ max and other outcomes (i.e., TTE, race time, RPE, peak power). When compared to a high carbohydrate diet, there are mixed findings for the effect of EAKD consumption on endurance performance. This may be partially due to the heterogeneity across studies and/or variability in athletes’ individual genetic factors, especially those that directly influence metabolism.

The limited number of published studies point to a need for more research in this field. Specifically, randomized studies performed in mixed sex samples are needed. Researchers might also consider examining EAKD-like diets that do not induce ketosis. Such research will expand our understanding of the diet’s effects in diverse athlete populations, all of whom serve to benefit from further knowledge, be the findings supportive of the diet or not.

## Data Availability

All data analyzed in this review are included in the following published articles. Burke, L.M., et al., *Low carbohydrate, high fat diet impairs exercise economy and negates the performance benefit from intensified training in elite race walkers.* J Physiol, 2017. **595**(9): p. 2785-2807.Carr, A.J., et al., *Chronic Ketogenic Low Carbohydrate High Fat Diet Has Minimal Effects on Acid-Base Status in Elite Athletes.* Nutrients, 2018. **10**(2). Heatherly, A.J., et al., *Effects of* Ad libitum *Low-Carbohydrate High-Fat Dieting in Middle-Age Male Runners.* Med Sci Sports Exerc, 2018. **50**(3): p. 570–579. McSwiney, F.T., et al., *Keto-adaptation enhances exercise performance and body composition responses to training in endurance athletes.* Metabolism, 2018. **81**: p. 25–34. Phinney, S.D., et al., *The human metabolic response to chronic ketosis without caloric restriction: Preservation of submaximal exercise capability with reduced carbohydrate oxidation.* Metabolism, 1983. **32**(8): p. 769–776. Shaw, D.M., et al., *Effect of a Ketogenic Diet on Submaximal Exercise Capacity and Efficiency in Runners.* Med Sci Sports Exerc, 2019. **51**(10): p. 2135–2146. Zinn, C., et al., *Ketogenic diet benefits body composition and well-being but not performance in a pilot case study of New Zealand endurance athletes.* J Int Soc Sports Nutr, 2017. **14**: p. 22.

## Abbreviations

EAKD: Endurance athlete’s ketogenic diet
VO_2_ max: Maximal oxygen uptake
TTE: Time to exhaustion
RPE: Rating of perceived exertion
